# Recent Advances in Nano-Drug Delivery Strategies for Chalcogen–Based Therapeutic Agents in Cancer Phototherapy

**DOI:** 10.3390/ijms26104819

**Published:** 2025-05-17

**Authors:** Subhrakant Jena, Abderrazzak Douhal

**Affiliations:** Departamento de Química Física, Facultad de Ciencias Ambientales y Bioquímica, and INAMOL, Universidad de Castilla-La Mancha, Avenida Carlos III, 45071 Toledo, Spain

**Keywords:** cancer therapy, chalcogen, nano-drug delivery, photodynamic therapy, photothermal therapy, reactive oxygen species, triplet harvesting

## Abstract

Chalcogen–containing therapeutic agents (TAs), which include sulfur (S), selenium (Se), and tellurium (Te) atoms, have recently emerged as a promising class of photosensitizers (PSs) and photothermal agents (PTAs) for cancer phototherapy. The incorporation of heavier chalcogens into organic chromophores leads to visible–to–near–infrared (VIS–NIR) light absorption, efficient triplet harvesting, and adequate heat and energy transfer efficiency, all of which are paramount for photodynamic therapy (PDT) and photothermal therapy (PTT). However, chalcogen–based PSs/PTAs suffer from photostability, bioavailability, and targeted delivery issues, which minimize their PDT/PTT performances. Nevertheless, significant progress in the rational design of nanoencapsulation strategies has been achieved to overcome the challenges of chalcogen–based TAs for effective phototherapeutic cancer treatment. This review highlights the recent advances (within the last five years) in nano-drug delivery approaches adapted for chalcogen–substituted PSs/PTAs for PDT, PTT, or synergistic PDT/PTT, integrating imaging and treatment. The PSs/PTAs described in this review are classified into three classes: (i) sulfur, (ii) selenium, and (iii) tellurium–containing TAs used in phototherapy applications. This review offers a comprehensive perspective on the design of chalcogen–substituted photosensitizers (PSs) and photothermal agents (PTAs), covering spectroscopic and computational characterization, nanoformulation strategies, and their roles in enhancing reactive oxygen species (ROS) generation and photothermal conversion efficiency for improved in vitro and in vivo performance. We hope this work will encourage further research into nanotechnological strategies designed to enhance the phototherapeutic efficacy of chalcogen–containing therapeutic agents.

## 1. Introduction

The chalcogens, including oxygen (O), sulfur (S), selenium (Se), and tellurium (Te), have been routinely incorporated into metal complexes [1,2], biomolecules [3,4,5], and organic chromophores [6,7,8] to access various applications. This chalcogen substitution approach has been thoroughly scrutinized in the context of unusual non-covalent interactions [9,10], improved magnetic characteristics [11,12,13], modified spectroscopic profiles [14,15,16,17], biomedical applications, etc. Particularly in biomedical applications, drugs or therapeutic agents (TAs) comprised of chalcogen elements have remarkable importance because of their chemical versatility and multi-disease therapeutic properties [18,19]. Chalcogen–based TAs exhibit a wide range of therapeutic properties, such as antioxidants, antimicrobials, ant–cancer, and other neurodegenerative diseases [18,19,20,21,22,23]. In recent times, the decoration of conventional organic chromophores with heavier chalcogen atoms (S, Se, and Te) has gained increased attention in phototherapy, i.e., the use of specific wavelengths of light to cure medical disorders [24,25,26,27,28].

The oldest form of phototherapy is heliotherapy, a thousand-year-old therapeutic approach that uses sunlight to treat skin diseases [29,30,31]. However, modern phototherapy began in the early 20th century when Niels Finsen developed a ‘chemical rays’ lamp that used blue- and red-light sources to treat skin diseases like cutaneous lupus vulgaris and smallpox, respectively [32,33,34]. In 1903, Niels Finsen was awarded the Nobel Prize in Physiology or Medicine [35]. Over the past century, modern phototherapy has advanced significantly, offering novel treatments for infections, cancer, and skin diseases [36,37,38]. Among the various forms of phototherapy, photodynamic therapy (PDT) and photothermal therapy (PTT) have emerged as promising alternatives to conventional treatments such as surgery, radiotherapy, chemotherapy, and immunotherapy. They offer less invasive strategies, high selectivity, and minimal damage to normal tissues [38,39].

**Scheme 1:**
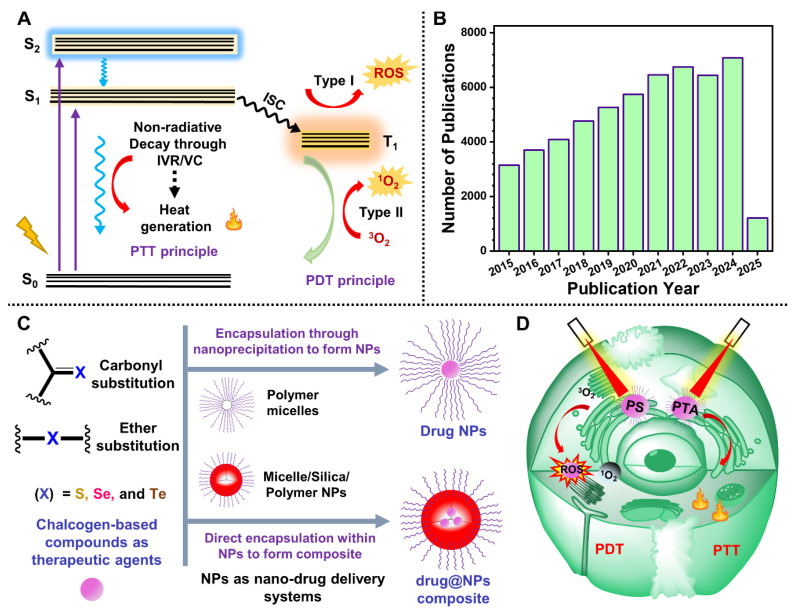
(**A**) A simplified Jablonski energy diagram representing the main photophysical and photochemical processes involved in PDT and PTT. ISC: intersystem crossing, IVR: intramolecular vibrational energy redistribution, and VC: vibrational cooling. (**B**) Increase in the number of publications from 2015 to present, obtained from the search for phototherapy (with keywords PDT and PTT) in the Web of Science portal (https://www.webofscience.com/wos/woscc/basic-search, accessed on 12 March 2025). (**C**) Illustration of the design and nanoformulation strategies for chalcogen–based conventional organic chromophores. (**D**) Schematic representation of the cell apoptosis process induced by PDT/PTT upon irradiation of nanoformulated TAs.

Scheme 1A illustrates a simplified Jablonski diagram outlining the mechanism of PDT and PTT. In the former, the photosensitizers (PSs) in the presence of molecular oxygen (O_2_) absorb light of appropriate wavelength and transition to electronically excited singlet states (S_1_ or S_2_), followed by intersystem crossing (ISC) to a long–lived triplet excited state (T_1_). In a type–I reaction, the triplet PS interacts with cellular substrates via electron transfer, generating reactive oxygen species (ROS) such as hydroxyl radical (OH•), hydrogen peroxide (H_2_O_2_), and superoxide anion (O_2_•^−^). Alternatively, in a type–II reaction, triplet PS engages by transferring its energy to the triplet ground state of O_2_ and produces reactive singlet oxygen (^1^O_2_) [40]. In PTT, photothermal agents (PTAs) absorb near–infrared (NIR, 750–1350 nm) light, relax non-radiatively via intramolecular vibrational energy redistribution (IVR) or vibrational cooling (VC), and dissipate energy in the form of heat [41]. This localized hyperthermia (50–100 °C) is characterized by photothermal conversion efficiency (PCE). Both ROS (free radicals and ^1^O_2_) and hyperthermia contribute to cellular apoptosis.

Scheme 1B shows the number of publications on synergistic phototherapy (PDT and PTT) in the last 10 years (2015 to present), highlighting unprecedented progress in phototherapeutic treatment for developing various PSs, PTAs, and light sources. Chalcogen compounds are one of the promising classes of TAs that possess the photophysical properties required for ROS production and PCE [28]. The incorporation of heavier chalcogen atoms varies, including the endo and exocyclic functionalization of the aliphatic and aromatic backbone of organic molecules containing ether, carbonyl, and other functional groups [26,27,28,42,43]. Chalcogen (S/Se/Te)–substituted compounds help in achieving the necessary criteria to design ideal PSs/PTAs. The rationale behind chalcogen incorporation includes the following:

1. Sustainable synthesis: Chalcogen incorporation facilitates more efficient, economically viable synthetic strategies with high product yield and reproducibility, essential for scalable biomedical applications.
2. Enhanced optical absorption: The substitution of heavier chalcogens extends the visible to NIR (VIS–NIR) absorption by lowering the highest occupied molecular orbital (HOMO) and the lowest unoccupied molecular orbital (LUMO) energy gap and enhancing the intramolecular charge transfer (ICT) character.
3. Improved ROS generation: The presence of heavier chalcogens enhances the spin-orbit coupling (SOC) constant due to the heavy–atom effect and narrows the singlet to triplet energy gap (ΔE_ST_) to facilitate ISC, leading to high triplet harvesting and ROS production, which is crucial for PDT.
4. Elevated PCE: Chalcogen substitution triggers ICT and other nonradiative processes, resulting in increased PCE, a fundamental property for effective PTT.
5. Biocompatibility: Chalcogen–containing compounds or TAs demonstrate superior in vivo biocompatibility and lower dark cytotoxicity, thereby improving the biological window for synergistic therapy.

However, chalcogen–based TAs often exhibit low aqueous solubility, which adversely affects their bioavailability compared to conventional organic molecules. This limitation hinders their effective delivery to specific biological targets. Moreover, in phototherapy applications, the low photostability of chalcogen compounds can lead to rapid photodegradation and undesired photochemical reactions, potentially causing side effects and restricting their therapeutic potential [18,26,27,44,45,46].

Nevertheless, nanoparticles (NPs) have emerged as promising nano-drug delivery systems for TAs, effectively addressing several challenges associated with conventional drug delivery, including poor aqueous solubility, limited bioavailability, and a lack of targeting ability [47,48,49,50]. These advantages are primarily achieved by the nanoencapsulation of TAs, wherein NPs function as protective nanocarriers. Nanocarriers protect the TAs from premature enzymatic and metabolic degradation, extend circulation time, and improve bioavailability. Furthermore, the physicochemical properties of NPs, such as their tunable size and surface chemistry, critically influence their biodistribution, stability, and cellular uptake, thereby enabling targeted and controlled release at the desired site for in vivo activity. A wide range of nanomaterials, including lipid–based NPs, polymeric NPs, inorganic NPs, and many others, have been employed for drug encapsulation [49,51,52].

Considering their advantages, significant efforts have been devoted to chalcogen–based TAs and their nano-drug delivery systems, aiming to enhance phototherapeutic applications [24,25,26,27]. Nanoformulation strategies include the use of lipid–based, micellar, silica, and polymer NPs to improve the in vitro/in vivo efficacy. Scheme 1C illustrates the two majorly adapted nanoformulation strategies to fabricate chalcogen compound–based NPs: (i) the formation of drug NPs by encapsulating TAs using polymer micelles through the nanoprecipitation method, and (ii) the formation of drug@NPs through the direct encapsulation of the TAs within nanocarriers using the self-assembly process. These strategies address the solubility issue, maintain colloidal stability, and improve photostability and targeting ability. Scheme 1D displays the light-induced PDT/PTT process in a representative cell treated with nanoformulated TAs (PSs and PTAs). The generation of ROS and/or heat leads to cancer cell apoptosis through protein denaturation or damage to the cell membranes and other organelles [53,54,55].

Numerous review articles have been dedicated to the nano-drug delivery systems for phototherapeutic agents [56,57,58,59]. The impact of the exocyclic and endocyclic substitution of chalcogens in conventional organic chromophores for phototherapy applications has been recently reviewed [28]. A combined effort encompassing synthesis, photophysics, and cellular studies further underscores the interdisciplinary nature of this research field. However, to our best knowledge, no review has focused on the recent advances in the nanoformulation of chalcogen–substituted TAs for synergistic PDT/PTT. Thus, in this review, we emphasize the crucial role of the nano-encapsulation of chalcogen–substituted TAs in cancer phototherapy by summarizing the advances made during the last five years. We also provide basic concepts of PDT and PTT, computational and spectroscopic characterization, and the role of the nanoformulation of chalcogen–based TAs in boosting phototherapeutic efficiency. In addition to the introduction and conclusion, this review is structured into three sections. Each one is dedicated to TAs containing one chalcogen atom–, starting with S and following with Se and Te elements, with subsections discussing the crucial role of nanoformulation in PDT, PTT, and imaging–guided synergistic PDT/PTT applications. At the end of this review, we summarize the key findings and suggest potential directions for advancing current understanding and developing smarter tools. We anticipate that this review will stimulate further research in the field and encourage young researchers to join the scientific community focused on engineering nanocarriers for the efficient delivery of chalcogen–based TAs.

## 2. Advances in Nano Delivery Strategies for Sulfur–Containing TAs in Phototherapy

S is one of the earliest known elements used in therapeutic applications since ancient times [60]. S–containing compounds display antibacterial, antimicrobial, antiviral, and cytotoxicity therapeutic properties and have vast applications in the pharmaceutical industry [18,61,62]. The incorporation of S has been further extended into heterocycles, metal complexes, and conventional chromophores for phototherapy applications. This has been achieved through exo/endocyclic S substitution through different functional groups of chromophore units. It leads to red–shifted absorption and increased ROS production, crucial for PDT and PTT applications. In this section, we discuss the performance of S-containing TAs and the role of nanoformulation in augmenting the in vitro/in vivo PDT/PTT efficiency.

### 2.1. Thiocarbonyl–Based PSs in Phototherapy

The S atom substitution in the exocyclic carbonyl oxygen of nucleobases, conventional organic chromophores, is one of the novel approaches in the design of heavy–atom–free PSs (HAFPSs) for chemo and phototherapeutic cancer treatment [63,64,65,66,67,68]. Thiocarbonyl modification red shifts the maximum of absorption intensity toward the optimal therapeutic window and promotes ISC for efficient ROS generation, which is paramount for PDT. Recent studies also focused on the use of thiocarbonyl–based HAFPSs for PTT [69,70,71]. In the following subsection, we focus on the advances in the nano-drug delivery of thiocarbonyl–based TAs with selected examples in the context of PDT, PTT, or synergistic PDT/PTT.

#### 2.1.1. Northiosquaraine–Based PSs in PDT

Squaraine dyes are a class of zwitterionic molecules with an electron–deficient four-membered ring derived from squaric acid and can be functionalized with electron-donating groups to form a donor–acceptor (D–A) skeleton. Moreover, the encapsulation of squaraine dyes within NPs can form H– or J–aggregates due to robust intermolecular interactions, causing a blue or red shift in the absorption intensity maximum, respectively [72]. Therefore, squaraine dyes exhibit interesting photophysical properties that have been extensively scrutinized in phototheranostic applications [73].

**Figure 1:**
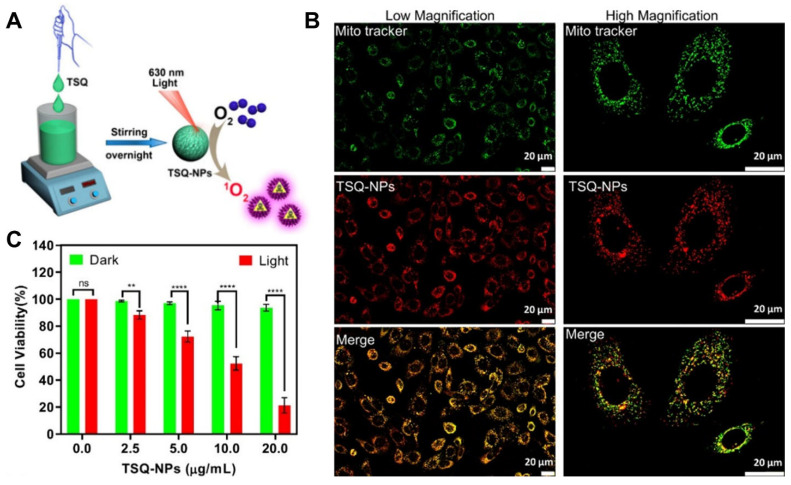
(**A**) Illustration of the preparation and applications of TSQ-NPs. (**B**) Fluorescence microscope image of TSQ-NPs on labeled KB cells showing subcellular accumulation of TSQ. The low and high magnification images of KB cells utilize blue excitation for Mito–Tracker green (green emission) and green excitation for TSQ (red emission). The scale bar indicates 20 μm. (**C**) TSQ-NPs induced an in vitro cytotoxic effect (20 μg/mL) against KB cells upon irradiation using red light (6 W, λ = 630 nm) for 5 min. * Data from ≥2 replicates. ns, not significant. Adapted with permission from Ref. [68]. Copyright 2024, American Chemical Society.

In 2024, the phototheranostic activity of northiosquaraine (TSQ)–based NPs was reported for image–guided PDT [68]. Figure 1 highlights a few key findings of that contribution, as discussed below. Thio–carbonyl modification in the oxo-derivative (ISQ) was performed to synthesize TSQ. Figure 1A shows the formed TSQ Ps during the self-assembly process and a schematic demonstration of the used procedure and application. TSQ NPs exhibit a uniform size of around 50 nm, observed through field emission scanning electron microscopy (FESEM) measurements and verified by dynamic light scattering (DLS) studies.

The negative zeta potential of −19 mV of TSQ NPs helps to stabilize them within the biological medium through electrostatic repulsions. The TSQ NPs possess good water dispersibility, photostability, biocompatibility, and optical properties. The comparison of the absorption spectrum of TSQ with TSQ NPs confirmed the formation of J–aggregates in the aqueous solution of TSQ NPs. Upon light irradiation, an almost 60% decrease in the absorption intensity of ROS probe 1,3-diphenylisobenzofuran (DPBF) in 15 min confirmed ^1^O_2_ generation, which was further supported by the results of electron spin resonance (ESR) experiments. The ^1^O_2_ quantum yield (Φ_Δ_) of TSQ-NPs in water was 76%, which is higher than that of the TSQ (Φ_Δ_ = 54%) and is proposed to be among the highest reported organic PSs. The cellular uptake of TSQ-NPs was investigated under a fluorescence microscope by labelling KB and HeLa cells with TSQ-NPs. Figure 1B shows fluorescence microscope images of the Mito–Tracker green-based colocalization, which revealed the predominant mitochondrial localization of TSQ-NPs. The in vitro cell viability studies show cell death upon increasing the dose of TSQ-NPs and their exposure time to red light (Figure 1C. TSQ-NPs exhibit efficient ROS generation in response to deeply penetrating 630 nm red light and demonstrate good biocompatibility in the dark. The photocytotoxicity effect of TSQ-NPs on KB and HeLa cell lines further supports their potential PDT applicability.

#### 2.1.2. Thione–Derived Perylene Di–Imide–Based PTAs in PTT

Perylene di-imide (PDI)−based dyes are promising organic molecules with impressive thermal, chemical, and photostability properties routinely used in imaging–guided phototherapeutic applications [74,75]. In 2020, thiocarbonyl–modified PDI derivatives were reported as nanoagents for synergistic PDT/PTT applications [71]. In that study, the designed thionated cis (PDI−CS) and trans-isomer (PDI−TS) isomers were scrutinized to investigate the effect of thionation. The thionation of PDIs enhances ROS generation along with a higher PCE of 58.4%. Furthermore, in vitro/in vivo studies on human lung cancer (A549) cells showed better photothermal depression upon 660 nm light irradiation. Two years later, the PDI chromophore moiety was functionalized with phenyl substituents at the imide N site in combination with thionation to synthesize a series of thione products, 1S−PDI–D, 2S−*cis−*PDI–D, 2S−*trans*–PDI–D, 3S−PDI–D, and 4S−PDI–D, for PDT applications [76]. In this report, the 1S−PDI−D derivative with excellent ^1^O_2_ ability (Φ_Δ_ ~ 100%) and the highest two-photon absorption properties was selected for PDT applications. The time–dependent density functional theory (TD−DFT) and femtosecond transient absorption studies revealed an efficient ISC process mediated through strong SOC effects in the studied thione derivatives. The 1S−PDI−D derivative was strategically linked with the targeting peptide, FC131 (cyclo−[2−NaI−Gly−d−Tyr−Arg−Arg]), on both terminals and fluorescent cyanine5 dye and FC131 on each terminal for tracking purposes. The assembly size of 630−700 nm and disassembly of 5−15 nm of 1S−FC131 and Cy5−1S−FC131 in a buffer solution resolved the solubility problem of the 1S−PDI−D derivative under irradiation. In vivo experiments showed that the 1S−FC131 and Cy5−1S−FC131 are selective in recognizing A549 cells and possess promising antitumor activity in A549 tumor xenografted mice.

**Figure 2:**
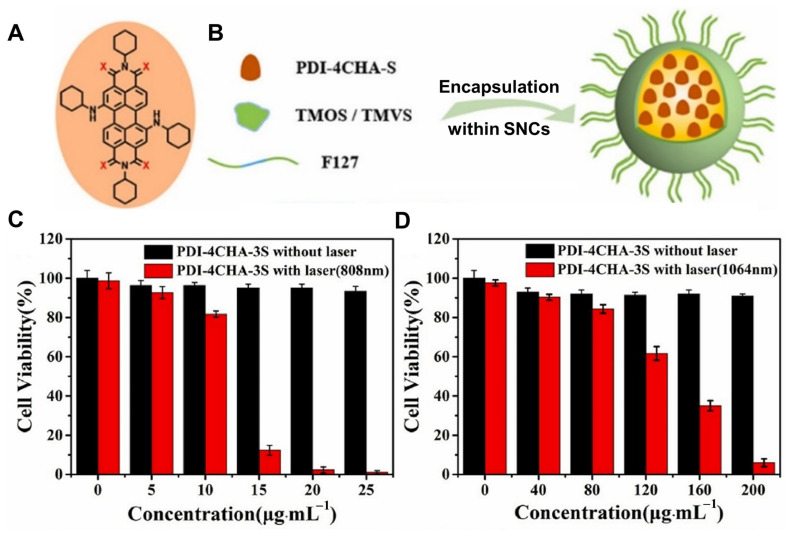
(**A**) Molecular structures of PDI−4CHA-X, where X = S. (**B**) Schematic representation of encapsulation strategy within silica nanocapsules (SNCs). (**C**,**D**) In vitro cell viability studies of 4T1 cancer cells incubated with PDI−4CHA−3S@SNCs at different concentrations with or without 808 nm and 1064 nm laser irradiation for 5 min (1.0 W cm^−2^). Adapted with permission from Ref. [69]. Copyright 2023, Elsevier.

However, the utilization of thiocarbonyl–modified organic chromophores was not explored in the context of PTT applications alone. In 2023, a series of thionated PDI (PDI−4CHA−S) derivatives was synthesized for superior PTAs [69]. The chemical structure of the thionated molecular skeleton is shown in Figure 2A. A site-selective S atom substitution and a symmetrical functionalization of two cyclohexylamines as electron–donating groups at the 1,7−bay positions of the PDI skeleton induced strong ICT and red shift in the absorption spectrum toward the NIR region. The PDI−4CHA−3S molecule with three S atoms exhibits an absorption intensity maximum at 854 nm, which was considered the highest reported to date for PDI derivatives. Though thiocarbonyl PSs are characterized by efficient triplet harvesting, an increase in the number of S atom substitutions in the PDI−4CHA skeleton decreases ROS yield, meaning no or very small contribution towards ISC. Considering the strong NIR absorbance intensity and low ROS yield, the photothermal effect was explored for PDI−4CHA−S derivatives. Figure 2B schematically presents the encapsulation of aggregated PDI−4CHA−S derivatives within silica nanocapsules (SNCs) to generate PDI−4CHA−S@SNCs composites.

The PDI−4CHA−S@SNCs were fabricated at different PDI−4CHA-S concentrations following the template hydrolysis method [77]. The encapsulation of PDI−4CHA−S molecules within SNCs was confirmed with transmission electron microscopy (TEM) and DLS experiments, suggesting good dispersity in aqueous solution. The steady–state absorption spectra of PDI−4CHA-S@SNCs with almost a 20 nm blue shift for each sample confirmed the formation of H-aggregates between PDI−4CHA−S molecules. The PDI−4CHA−3S@SNCs under 808 nm laser irradiation led to a maximum PCE of 88%. In addition, the PDI−4CHA−S@SNCs exhibit outstanding photothermal stability under repeated exposure to high-intensity 808 and 1064 nm laser irradiation. This illustrates the benefit of nanoencapsulation in overcoming the photobleaching effect. Overall, the PDI−4CHA−S@SNCs possess excellent water dispersibility, photostability, and bioavailability, making them suitable for biological studies. Furthermore, in vitro experiments on 4T1 murine cells revealed that tri–thionated PDI−4CHA−3S@SNCs show promising photocytotoxicity effects upon excitation at 808 and 1064 nm, as evident from Figure 2C and 2D. The high PCE in the NIR−II window (1064 nm) further showcases the crucial role of the exocyclic S atom substitution approach in designing PSs for PDT, but also PTAs for PTT.

Very recently, in 2025, the thionated PDI (1S−PDI) was encapsulated within the hydrophobic oily core of a lipid nanocapsule (LNC) to form a 1S−PDI@LNC composite with an encapsulation efficiency of 16% [78]. This resolved the aqueous solubility limitations of 1S−PDI and exhibited excellent photochemical stability. In phosphate buffer saline (PBS), 1S−PDI@LNC showed a promising ^1^O_2_ yield (Φ_Δ_ ~ 0.52), indicating efficient ROS generation. Notably, the in vitro and in vivo cytotoxicity studies revealed the low dark toxicity of 1S−PDI@LNC alongside enhanced photocytotoxicity effects. These findings underscore the potential of 1S−PDI@LNC as an efficient and targeted nanocarrier for PDT application.

#### 2.1.3. Thionated Porphyrin–Based TAs in Synergistic PDT/PTT

Porphyrins are an important class of organic molecules known for their low toxicity and good biocompatibility. They have garnered significant scientific interest due to their unique PDT and PTT effects integrated with excellent imaging capabilities [79,80]. The functionalization of its core moiety has been crucial in modulating the phototherapeutic properties. The development of nanotechnological approaches to improve water solubility and biocompatibility has also recently been documented for phototherapy applications [81].

**Figure 3:**
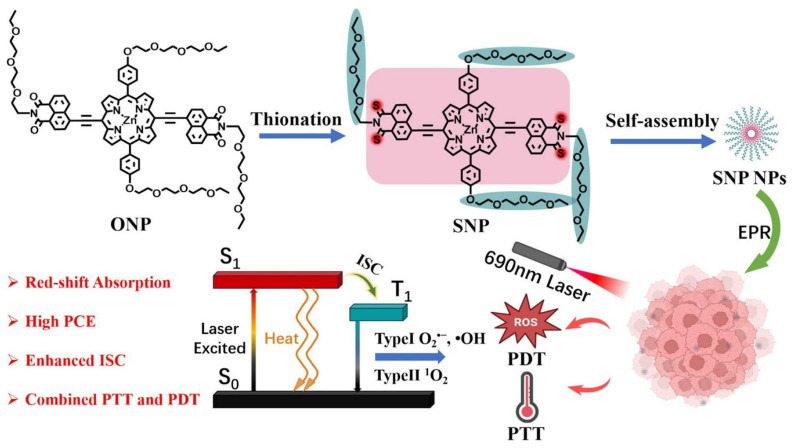
Illustration of the synthetic pathways of SNP from ONP and the self-assembly process to produce SNP NPs. It further demonstrates the irradiation of SNP NPs using 690 nm laser light to produce ROS with photothermal effects. The figure also depicts the relaxation processes responsible for PDT (type–I and type–II) and PTT (heat generation) and outlines the key involved photophysical properties. Adapted with permission from Ref. [70]. Copyright 2025, Elsevier.

In 2024, the chalcogen atom effect on the ISC rate of O and S-disubstituted heteroporphyrin was investigated using TD−DFT calculations. The results reveal enhancement in the SOC upon sulfur substitution in the porphyrin macrocycle, which further leads to efficient singlet–triplet ISC. The overall findings indicate an alternative approach to modulate the photophysical properties of porphyrins for PDT applications without incorporating heavy atoms [82]. Very recently, in 2025, the design and synthesis of two new organic porphyrin molecules (ONP and SNP) were performed with an acceptor–donor–acceptor (A−D−A) architecture for synergistic phototherapy, as summarized in Figure 3 [70]. The SNP was synthesized via the exocyclic thiocarbonyl modification of ONP. To enhance the aqueous solubility and photostability, the SNP was functionalized with polyethylene glycol (PEG) chains, resulting in the self–assembly of SNP NPs. The resulting nanomaterials exhibit spherical morphology with an average size of about 150 nm, confirmed through DLS and TEM measurements.

The SNP NPs resulted in enhanced hydrophilicity, photothermal stability, and biocompatibility. The ICT induced by A–D–A structures resulted in red-shifted NIR absorbance intensity maximum to 752 nm for SNP NPs and promoted non-radiative heat generation. The TD–DFT calculations further support the observed photophysical behaviors. The SNP and SNP NPs were observed to be non-fluorescent. The temperature of the SNP NPs solution increased by 20.2 °C when irradiated for 10 min at 150 µg/mL. In addition to that, S substitution in the exocyclic carbonyl position in the naphthalimide scaffold led to efficient ROS generation due to the pronounced SOC–mediated ISC process. Figure 3 also schematically illustrates the irradiation effect of SNP NPs with 690 nm laser light, producing PDT/PTT, and depicts the relaxation processes with some photophysical parameters. The synergistic (PDT and PTT) effects were reflected in the in vitro cytotoxicity studies against Hepa1-6 cells. The cell viability studies with SNP NPs without and with irradiation using 690 nm laser light revealed significant cell death due to the PDT/PTT effect. The authors proposed that the reported work demonstrates an efficient method to design porphyrin–based organic molecules and NPs for synergistic phototherapy.

### 2.2. Thiophene–Fused PSs/PTAs in Phototherapy

The incorporation of S through thiophene group functionalization is a recent strategy to modulate the photophysical properties of organic chromophores. Thiophene groups serve as donor groups and π–conjugated bridging moieties, and facilitate the ISC process in conventional organic chromophores for phototherapy applications [83,84,85,86]. In this section, we comment on the advances in nanoformulated thiophene-fused TAs in PDT, PTT, and synergistic PDT/PTT.

#### 2.2.1. Thiophene–Fused Osmium–Based PSs in PDT

The osmium quaterthiophene complexes exhibit pronounced light–triggered photocytotoxicity because of their extended wavelength near 655 nm and a ^1^O_2_ quantum yield of 95% [87,88]. In such complexes, the presence of an oligothiophene chain (four thiophene units) results in prolonged ^3^ILCT lifetimes, contributing towards high ROS production. These interesting photophysical properties result in light–triggered cytotoxicity toward cancer cells with normoxic EC_50_ values near 10 nM (633 nm red light) and as low as 20 pM for broadband visible light absorption under specific conditions (100 J cm^−2^ delivered at a rate of 20 mW cm^−2^) with very low dark toxicity. Further modification of the co–ligand in osmium quarterthiphene complexes has also been reported to be efficient for both hypoxia (1% O_2_) and normoxia (~18.5% O_2_) [88]. However, the presence of an oligothiophene chain in the osmium complex makes it prone to aggregation, and it remains water-insoluble by forming 1–2 μm–sized aggregates in the PBS. This intermolecular interaction could reduce potency and shows large variability between assays in the EC_50_ and phototherapeutic indices, with irreproducible in vitro activity.

**Figure 4:**
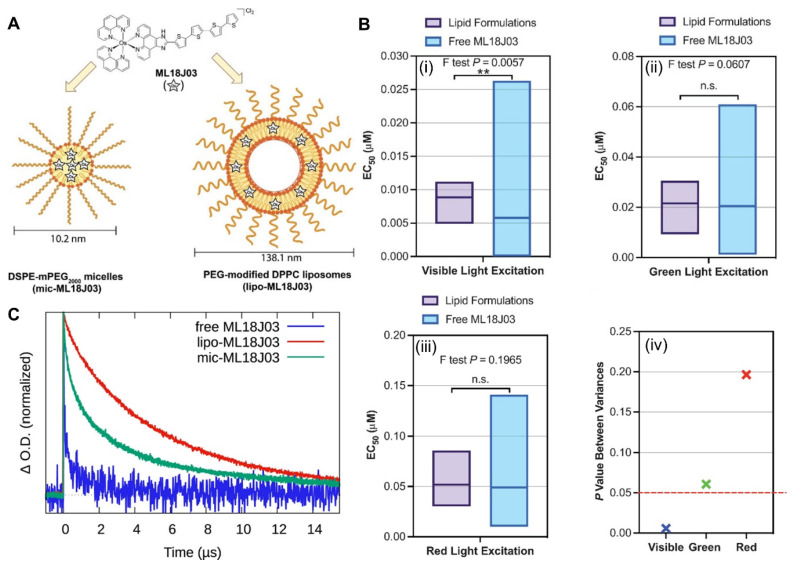
(**A**) Molecular structure of the Os–based PS rac–[Os(phen)_2_(IP–4T)](Cl)_2_ (ML18J03) and schematic representations of lipid formulations of ML18J03 using DSPE-mPEG2000 micelles (mic–ML18J03) and PEG–modified DPPC liposomes (lipo–ML18J03). (**B**) Min–max plots showing the interassay distribution of EC_50_ values of ML18J03 and lipid-formulated ML18J03 in SK–MEL–28 human melanoma cells upon irradiation using (**i**) Visible–light, (**ii**) Green–light, and (**iii**) Red–light, and (**iv**) Relationship between excitation light and statistical significance between variances demonstrating that the most reduced interassay variability is for visible–light excitation of lipid-formulated ML18J03. * Data from 2 replicates. ns, not significant. (**C**) Normalized transient absorption decays measured at 660 nm observation wavelength. Adapted with permission from Ref. [89]. Copyright 2022, John Wiley & Sons, Inc.

Recently, the lipid formulation of highly potent Os(II) PS, rac–[Os(phen)_2_(IP–4T)](Cl)_2_ (referred to as ML18J03) was reported, utilizing PEG–modified DPPC liposomes and DSPEmPEG2000 micelles, which could mitigate the aggregation issue, as depicted in Figure 4A [89]. The entrapment efficiency of PS within lipo–ML18J03 was 93%, while mic-ML18J03 achieved quantitative entrapment efficiency ranging from 84 to 100%, depending on the PS loading. The hydrodynamic diameters of lipo–ML18J03 and mic–ML18J03 were between 80–200 nm and 10.2–13.2 nm, respectively, both within the clinically acceptable range. The lipid nanoformulated ML18J03 exhibited excellent stability during 37 °C incubation and 4 °C storage. Furthermore, lipid nanoformulation enhanced the PS solubility and prevented aggregation, which was responsible for large interassay variability in phototherapeutic response. The results showed that the PDT efficacy was maintained in liposomes (lipo–ML18J03) and micelles (mic–ML18J03). Figure 4B depicts the min–max plot of EC_50_ values, which shows that the interassay variance reduced significantly for lipo–ML18J03 and mic–ML18J03 to unformulated ML18J03, under visible–, green–, and red–light excitation. In addition to that, the overall photophysical properties were maintained with lipid nanoformulation, whereas the kinetics remained altered.

Figure 4C displays the transient absorption decays for ML18J03, lipo–ML18J03, and mic-ML18J03. The lipo–ML18J03 and mic–ML18J03 exhibit longer ^3^ILCT lifetimes than those of free ML18J03 due to suppressed aggregation and are responsible for enhanced ^1^O_2_ production. The overall outcomes of the study show that the liposomal and micellar nanoformulation of osmium quarterthiophene complexes could overcome aggregation issues and might be useful for in vivo PDT.

#### 2.2.2. Thiophene–Fused Croconaine Dye–Based PTAs in PTT

Croconaine (CR) dyes represent a promising class of NIR–absorbing zwitterionic dyes with good photothermal and photoacoustic imaging (PAI) capabilities [90,91]. The croconic moiety of CR dyes with electron–withdrawing ability provides scope for functionalization with a large variety of electron donor groups to form donor–acceptor–donor (D–A–D) skeletons. This results in an extended π–conjugation system providing a narrow and intense absorption spectrum in the NIR region with a high molar extinction coefficient (∼10^5^ M^−1^ cm^−1^). However, they have been relatively less explored than their lower homologs, squaraine dyes for phototherapy applications.

**Figure 5:**
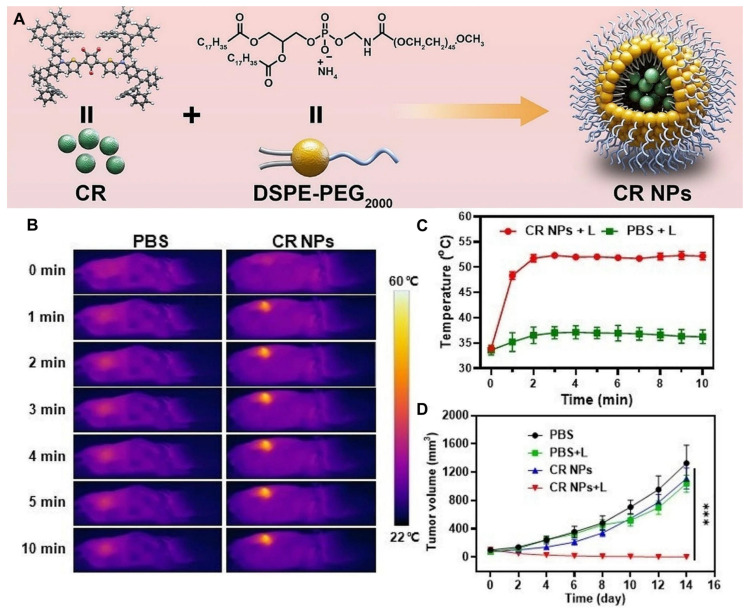
(**A**) Graphical representation of CR dye NP formation for tumor–targeting PTT. In vivo CR NP-mediated PTT. (**B**) IR thermal images, and (**C**) respective temperature changes at the tumor sites upon 808 nm (1.0 W cm^−2^) laser irradiation of Colon26 tumor-bearing mouse model treated with PBS or CR NPs at different time points (*n* = 5). (**D**) Tumor growth profiles of four groups during the treatment course. Data from ≥3 replicates. ns, not significant. *** *p* < 0.001. Adapted with permission under the terms of the CC-BY license from Ref. [92]. Copyright 2024, the authors, published by Springer Nature.

Recently, D–A–D structure–based croconaine dyes (CR–TPE–T) were synthesized and used in NIR–II fluorescence imaging (NIR–II FLI) PTT [92]. Figure 5 highlights several noteworthy findings of the study, which we discuss in detail below. First, the TD–DFT calculations and molecular orbital visualization of CR–TPE–T revealed the electron density in HOMO primarily located at the tetraphenylethylene (TPE) donor along its thiophene conjugated units. In contrast, the electron density in LUMO is localized on the electron–deficient croconaine core.

This electron density distribution facilitates efficient ICT within the molecule. This leads to extended absorption in the NIR–I region with a computed energy band gap of approximately 1.45 eV. Figure 5A shows the encapsulation of the hydrophobic CR–TPE–T molecule within the DSPE–mPEG2000 matrix through the nanoprecipitation method to fabricate CR NPs for in vivo applications. The encapsulation efficiency was 93%. The TEM images and DLS studies confirmed the spherical shape of the CR NPs with an average diameter of ~158 nm. The zeta potential of CR NPs was −33.7 ± 1.71 mV and consistent diameter distribution after 5 weeks of storage at room temperature, confirming excellent colloid stability in physiological conditions. These findings highlight the potential of CR NPs for NIR–II FLI–guided PTT applications. The CR NPs displayed an intense absorption peak at 870 nm, which extended to over 1000 nm. The CR NPs showed an emission maximum of 970 nm and extended to over 1200 nm (NIR–II), with a quantum yield of 0.47% and PCE of 65% upon 808 nm irradiation. In vivo experiments were performed in a colon26 tumor-bearing mouse model for NIR–II FLI PTT. Figure 5B and 5C illustrate the tumor region’s temperature through the real-time IR thermal images and the change in the temperature with time in the presence of PBS and CR NPs upon 808 nm laser irradiation. Figure 5D shows the tumor growth profile during treatment, demonstrating a complete inhibition of the tumor, improving the survival of mice, inducing efficient immune responses, and reducing lung metastasis. This promising in vivo performance further signifies the pronounced photothermal therapeutic efficiency of CR NPs. The authors reported the first-ever study in the design of small organic molecule-based PTAs from croconaine dyes for NIR–II–FLI–guided PTT applications.

#### 2.2.3. Thiophene π–Bridge Manipulation–Based TAs in Multimodal Synergistic PDT/PTT

**Figure 6:**
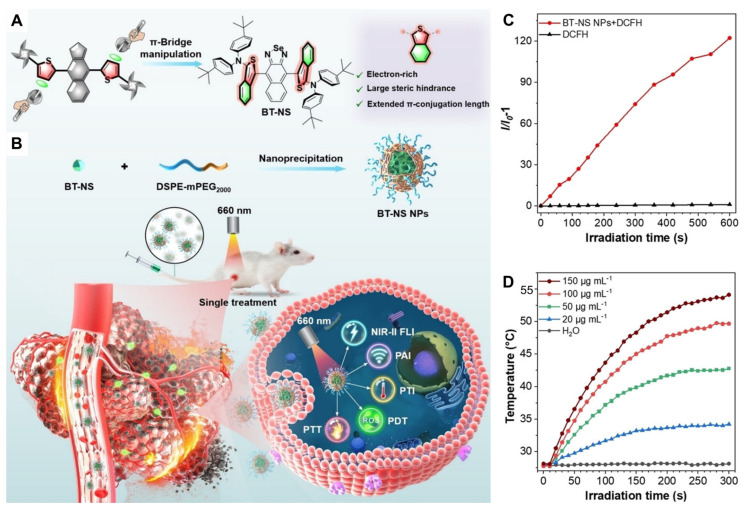
Schematic demonstration of π–bridge engineered NIR–II AIEgens with multimodal phototheranostic performance. (**A**) Illustration of π–bridge manipulation strategy for constructing NIR–II AIEgens. (**B**) Illustration of the nanofabrication process of BT-NS to form BT-NS NPs and its application in FLI/PAI/PTI trimodal imaging–guided synergistic phototherapy. (**C**) ROS production ability of BT-NS NPs (5 μM) upon 660 nm laser irradiation (0.3 W cm^−2^). (**D**) Temperature change in BT-NS NPs at varied concentration in aqueous solutions upon 660 nm laser irradiation (0.3 W cm^−2^). Adapted with permission from Ref. [93]. Copyright 2024, John Wiley & Sons, Inc.

Recently, the effect of thiophene π–bridge manipulation was reported in D–A based NIR-II aggregation-induced emission luminogens (AIEgens) for FLI/PAI/photothermal imaging–guided synergistic PDT/PTT, as shown in Figure 6 [93]. In that work, the functionalization of benzo[c]thiophene moiety was performed to synthesize TNS, C6T-NS, OT-NS, and BTNS AIEgens as shown in Figure 6A. Quantum chemical calculations revealed that the BT-NS exhibits a decreased energy band gap of 1.74 eV, signifying a red–shifted absorption wavelength ascribed to the increased conjugation length of the benzo[c]thiophene unit.

UV–Vis–NIR absorption and photoluminescence spectroscopies showed that the BT-NS molecule has the most red–shifted intensity maxima of absorption and emission at 660 and 1003 nm, respectively, among the studied molecules. BT-NS displayed AIE characteristics with a high PCE because of a strong ICT process resulting from thiophene π–bridge manipulation and ROS production capacity due to the presence of the Se atom. The authors encapsulated BT-NS within DSPE–mPEG2000 as a matrix to form uniform NPs and investigated the phototheranostic ability, as illustrated in Figure 6B. DLS and TEM studies revealed that the size of the fabricated BT-NS NPs is around 67 nm, optimal for tumor site accumulation, enhanced permeability, and retention effect. BT-NS NPs also possess outstanding colloidal stability in water, verified through size and absorption measurements. Figure 6C displays the 122–fold enhancement in the ROS production capacity of BT-NS NPs, showcasing the potential utilization of these NPs in PDT. As evident from Figure 6D, the change in temperature of BT-NS NPs depends upon sample concentration and laser power density, with a calculated PCE of 41.8%. The in vitro and in vivo experiments on 3T3 cells and 4T1 tumor-bearing mice were successfully conducted to eliminate the tumors using BT-NS NPs through imaging–guided synergistic PTT/PDT. Moreover, in the absence of light, BT-NS NPs exhibited excellent cell viability, underscoring minimal dark cytotoxicity and excellent biocompatibility. These findings further elucidate the key role of thiophene moiety regulation, which can effectively tweak the molecular backbone for improved phototherapy.

To summarize this subsection, S is the most extensively studied chalcogen element in PDT, PTT, and synergistic PDT/PTT applications. Its substitution has been performed in several organic molecules through exocyclic carbonyl, exo/endocyclic ether oxygen replacement, and the functionalization of various functional groups. As discussed above, thiocarbonyl and thiophene groups are frequently integrated into organic chromophores such as PDIs, squaraines, croconaines, BODIPYs, porphyrins, and their metal complexes. The incorporation of S through these groups enhances ICT and SOC events, which are essential for VIS–NIR absorption and triplet harvesting, thus leading to improved PCE and ROS yield. Additionally, manipulating the thiophene groups allows for NIR–FLI–guided therapeutic potential. The nanoformulated S–containing TAs demonstrate favorable size and water dispersibility, photostability, and biocompatibility in aqueous solutions. These result in precise control and enhanced in vitro and in vivo PDT, PTT, and synergistic imaging-guided PDT/PTT efficiency.

## 3. Advances in Nano Delivery Strategies for Selenium–Containing TAs in Phototherapy

Se is an essential trace element in the human body and participates in various biochemical processes [18,19,94]. It acts as an antioxidant at low concentrations, and its toxicity depends on the Se derivative used for medical administration [95]. It is well known that Se and Se–containing compounds possess antioxidant, anticancer, and chemopreventive activities [18,22,23,96,97]. The Se atom, being heavier, with less electronegativity than the S one, can trigger ICT and SOC effects when incorporated into organic chromophores. The exo/endocyclic incorporation of Se into heterocycles and conventional chromophores results in an extended VIS–NIR absorption spectrum due to the pronounced ICT effect and ROS production. The combined ICT and ISC processes are more effective in Se derivatives than S ones and show improved PDT and PTT applications. In this section, we discuss the performance of Se–containing TAs and the role of nanoformulation in overcoming the problems faced by them to improve the in vitro/in vivo PDT/PTT performance.

### 3.1. Benzoselenadiazole–Containing PSs in Type–I PDT

**Figure 7:**
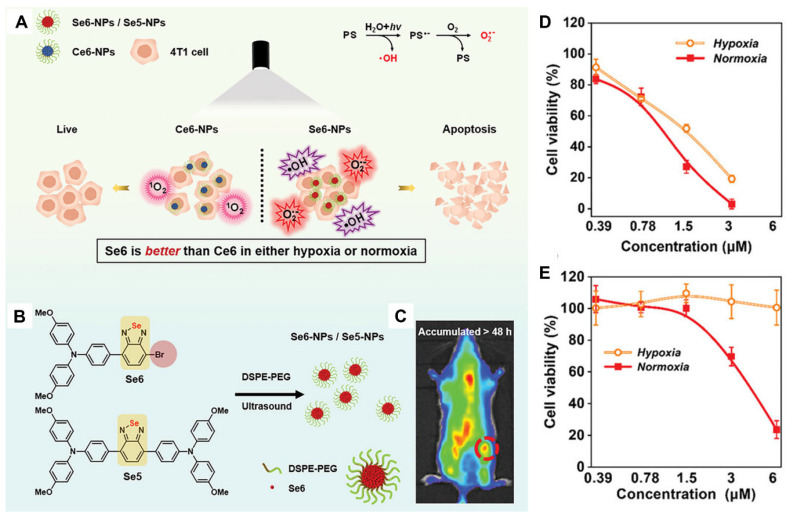
(**A**) Illustration of type–I PDT processes of Se6-NPs and type–II PDT processes of chlorin e6 (Ce6)-NPs: Se6-NPs exhibited better PDT treatment than Ce6-NPs in either hypoxia or normoxia conditions by generating dual radicals (^•^OH and O_2_^•−^). The proposed radical generation processes were also shown in the case of Se6-NPs in an aqueous solution (top right corner). (**B**) Chemical structures and NPs of Se6, Se5, and amphiphilic copolymer DSPE–PEG. Cell viability studies of 4T1 cells treated with (**C**) Se6-NPs and (**D**) Ce6-NPs at different concentrations upon 488 nm light irradiation (20 mW cm^−2^) for 30 min under normoxia or hypoxia conditions (*n* = 6, mean ± SD). (**E**) Picture showing the accumulation of Se6-NPs for more than 48 h in the tumor sites of a tail vein-injected tumor-bearing mouse model. Adapted under the terms of the CC–BY license from Ref. [100]. Copyright 2023, the authors, published by John Wiley & Sons, Inc.

Se atoms are incorporated in organic chromophores through donor– or acceptor–based heterocyclic groups containing them to design NIR PSs for PDT. Specifically, benzoselenadiazole is such a unit that exhibits a stronger electron-withdrawing ability than the S one used to derivatize with different electron-donating chromophoric moieties [98,99]. The resulting D–A skeleton holds strong ICT effects and imposes a stronger heavy–atom effect than the S atom, promoting ISC for a triplet–state generation. The combined effect of Se incorporation leads to an extended NIR absorption spectrum and triplet-state generation, crucial for PDT application. Recently, Se–containing D–A molecules (Se6, Se5) with AIE properties were designed for type–I PSs for PDT, as summarized in Figure 7 [100]. Figure 7A illustrates the involved type–I PDT process of Se6 and Se5 NPs with better PDT efficiency than Ce6-NPs under either hypoxia or normoxia conditions. The replacement of S with Se in the benzothiadiazole acceptor increases the ICT effect and results in a red–shifted absorption band (400–600 nm) for Se6 and Se5 molecules, and the results are consistent with the computed HOMO–LUMO gap. The presence of an additional bromine atom (Br) in the Se6 molecule doubles the heavy–atom effect and reduces the ΔE_ST_ to as small as 0.03 eV, which in turn promotes ISC and triplet–state generation. 

Figure 7B displays the chemical structure of Se5 and Se6 molecules, which were encapsulated inside a DSPE–PEG2000 amphiphilic copolymer and fabricated through the nanoprecipitation method. The resulting Se6 and Se5 NPs exhibited spherical nanostructures with an average hydrodynamic diameter of 115 and 87 nm in water, confirmed by SEM and DLS experiments. Additionally, Se6 and Se5 NPs displayed good water dispersibility and stability in blood. Due to the π–π interaction in their aggregates, the formed Se6 and Se5 NPs exhibited red–shifted absorption peaks around 550 and 518 nm, respectively. The type–I ROS production was confirmed with the help of ROS indicators and ESR measurements. The light-triggered ROS production efficiencies of Se6 NPs were observed to be notably higher than those in the case of Se5 NPs. Figure 7C,D show the cell viability of 4T1 cells incubated with Se6 and commercial chlorin e6 (Ce6) NPs, respectively, under normoxic and hypoxic conditions. The results indicated that the Se6 NPs display superior in vitro PDT performance than that of Ce6 NPs. Figure 7E displays the in vivo fluorescence images of effectively accumulated Se6 NPs after intravenous injection into 4T1-tumor-bearing BALB/c mice. The in vivo PDT performance revealed that the Se6 NPs exhibit 99% tumor growth inhibition rates, higher than that of the commercial Ce6 NPs. The overall finding of the study offers new insights into the design of type–I PSs.

### 3.2. Heptamethine Cyanine PSs in NIR PDT

Se atoms are also incorporated in organic dyes to design NIR PSs for PDT in deep tissues. Specifically, the Se triggers strong ICT effects and imposes a stronger heavy–atom effect, ensuring effective NIR photon capture and efficient triplet-state generation, which is crucial for deep–tissue PDT application. Notably, cyanine dyes are widely used in phototherapy applications among the available dye scaffolds due to their modifiable skeleton and excellent photophysical properties [101]. Heptamethine cyanine is one of the cyanine dyes that has recently received extensive research interest among the scientific community due to its cancer theranostics applications [102].

**Figure 8:**
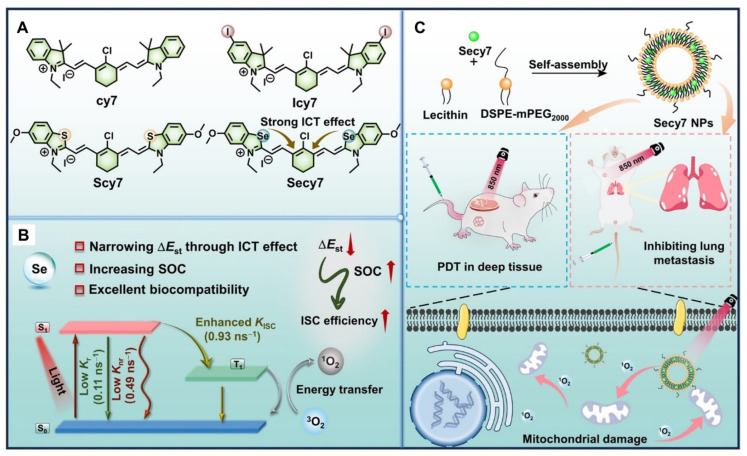
(**A**) The chemical structure of the investigated cy7 derivatives. (**B**) Se facilitated relevant photophysical parameters to enhance the ISC process, leading to ^1^O_2_ generation. (**C**) Illustration of self–assembly process for Secy7 NPs production and demonstration of in vivo PDT in a mouse model. Reproduced with permission from Ref. [103]. Copyright 2024, John Wiley & Sons, Inc.

Recently, in 2024, the use of heptamethine cyanine–based NIR PSs was investigated for anticancer PDT, as summarized in Figure 8 [103]. In that report, iodine (I), S, and Se atoms were systematically incorporated in the heptamethine cyanine (cy7) scaffolds to synthesize Icy7, Scy7, and Secy7, respectively, as shown in Figure 8A. The synthesized cy7 derivatives exhibited NIR absorption, with Secy7 displaying the highest red–shifted absorption intensity maximum, ~840 nm. The strong ICT effects between the Se atom and polymethine chain significantly lower the HOMO–LUMO energy gap of 0.51 eV and corroborate well with experimental steady–state absorption data. Remarkably, the ^1^O_2_ generation yield for Secy7 was 24.5–fold that of indocyanine green (ICG, reference). Femtosecond (fs) transient–absorption experiments revealed the excited-state dynamics of cy7 derivatives and the photophysical processes involved during electronic relaxation, as depicted in Figure 8B.

In addition to strong ICT effects, the heavy atom effect from Se atoms strengthens SOC and narrows the ΔE_ST_ with a maximum SOC of 1.4 cm^−1^ and a triplet–state yield of 61% for Secy7. Figure 8C shows the self-assembly process of Secy7 inside DSPE–PEG2000 and lecithin as carriers to generate Secy7 NPs, which could enhance the bioavailability and accumulation within the solid tumors for successful in vivo demonstration. Secy7 NPs exhibited spherical morphologies with an average diameter of 54.46 nm, as revealed by SEM and DLS analyses. Moreover, minimal changes in the size and absorbance of Secy7 NPs after 7 days in water demonstrate their good colloidal stability. Secy7 exhibited a notable tumor inhibition rate of 97% upon irradiation with 850 nm light in BALB/c mice bearing subcutaneous 4T1 tumor cells. Secy7 NPs also showed pronounced phototoxicity towards cancer cells buried under 12 mm of tissue. The overall results demonstrated the remarkable potential of Secy7 for advancing PDT in deep tissues and a step forward in the design of NIR PSs with ultrahigh ^1^O_2_ quantum yield.

### 3.3. Conjugated Small Molecule–Based PTAs in NIR–II PTT

**Figure 9:**
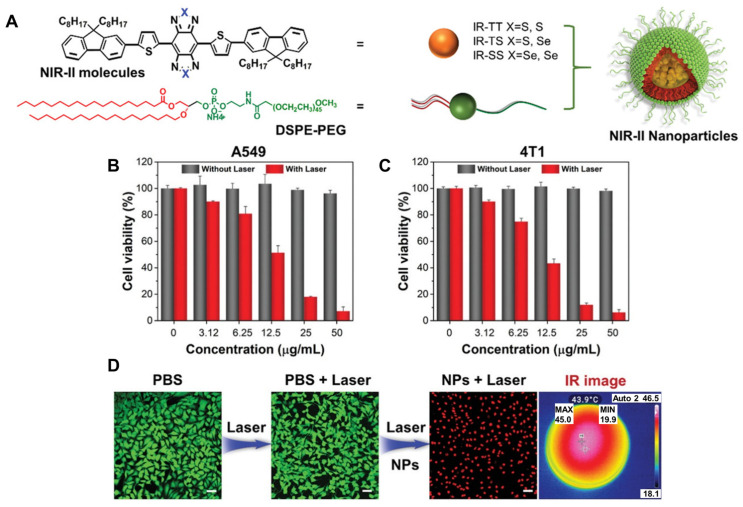
(**A**) Schematic illustration of the preparation process of the NIR–II NPs. Cytotoxicity of (**B**) A549 cells and (**C**) 4T1 cells incubated with IR–SS NPs of various concentrations with or without 1064 nm laser irradiation (1 W cm^−2^). (**D**) Live/dead confocal laser scanning images of A549 cells after multiple treatments. Green fluorescence calcein AM and red fluorescence PI represent live and dead cells, respectively. The scale bar represents 50 μm. Adapted with permission from Ref. [24]. Copyright 2020, John Wiley & Sons, Inc.

Previously, NIR–II PTAs were either inorganic or polymeric materials, whereas biocompatible small-molecule PTAs were limited to the NIR–I bio-window [104,105]. Introducing ICT effects within small organic molecules is thus of great importance for improved photothermal conversion in small-molecule PTAs. In 2020, chalcogen atoms were strategically incorporated to design a family of conjugated oligomer species (IR–TT, IR–TS, and IR–SS), each containing a D–π–A–π–D framework for superior NIR–II PTT, as summarized in Figure 9 [24]. Figure 9A illustrates the progressive substitution of S and Se atoms in the benzo [1,2c:4,5-c’]bis([1,2,5]thiadiazole acceptor unit. The thiophene groups act as π–conjugation units and electronic bridges between D–A–D to promote ICT events. Subsequent heavier chalcogen substitution (S replaced with Se) lowered HOMO–LUMO energy gaps, shifting the absorption peaks from 830 nm (IR–TT) to 930 nm (IR–TS) and 1060 nm (IR–SS). The resulting molecules were self-assembled into NPs and encapsulated with amphiphilic copolymer DSPE–PEG2000 to enhance stability and prolong blood circulation with an encapsulation yield of 88%. All the fabricated NPs exhibited a uniform spherical shape with an average hydrodynamic diameter of ~100 nm, measured by SEM and DLS experiments. Also, all the NPs possessed good dispersity in different media such as pure water, PBS, Dulbecco’s modified Eagle medium, and 10% fetal bovine serum (FBS), displaying their excellent stability.

The formed IR–TT, IR–TS, and IR–SS NPs exhibited a PCE of about 61%, 73%, and 77%, respectively. Figure 9B,C show the cell viability of A549 and murine breast cancer (4T1) cells treated with IR–SS NPs in the dark and 1064 nm laser irradiation, confirming the photothermal-induced killing of cancer cells in a dose–dependent manner. Figure 9D displays the confocal laser scanning microscope images of A549 cells upon 1064 nm laser irradiation. The visualization of red fluorescence in the presence of IR–SS NPs indicates a complete photoablation of cancer cells. Interestingly, the IR–SS NPs exhibited excellent PAI signal and tissue penetration depth. In vivo experiments, in tumor–xenografted mice, showed a rapid hyperthermia (~34 to ~63 °C) under 5 min 1064 nm laser irradiation, showing a complete inhibition of tumor growth in the presence of IR–SS NPs. This illustrates the photothermal capability of IR–SS NPs with a promising PTT effect, biosafety, and high-resolution, precise photoacoustic imaging in the NIR–II window.

### 3.4. Single–Atom Engineered Hemicyanine–Based TAs in Imaging–Guided Synergistic PDT/PTT

Incorporating Se atoms in NIR dyes has also been employed in multimodal phototherapy. As discussed previously, the combined ICT and heavy–atom effects are responsible for extended NIR absorption/emission, leading to the photothermal effects and ROS production. Hemicyanine dyes are one important class of cyanine dyes extensively used as NIR fluorescence reporters for targeted anticancer drug delivery, monitoring, and imaging applications [106].

**Figure 10:**
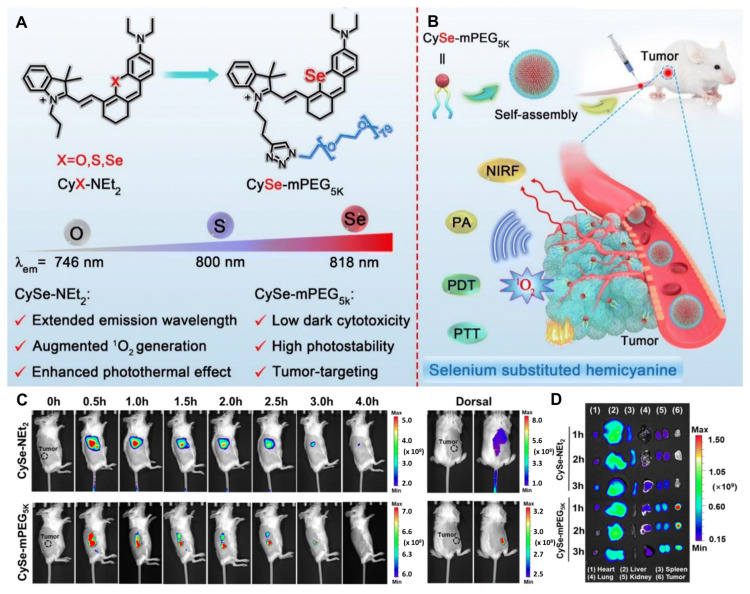
(**A**) Molecular structures of CyX–NEt_2_ (X = O, S, Se) and CySe–mPEG_5K_, designed through the copper(I)–catalyzed click reaction of CySe–NEt_2_ and a PEG chain. (**B**) Schematic demonstration of tumor-targeting imaging–guided multimodal phototherapy. (**C**) In vivo fluorescence imaging in tail vein–injected tumor-bearing mice treated with CySe–NEt_2_ and CySe–mPEG_5K_ (100 mM, 100 mL in PBS buffer). (**D**) In vivo biodistribution fluorescence images of tail vein-injected tumor-bearing mice treated with CySe–NEt2 and CySemPEG_5K_ for 1 h, 2 h, and 3 h. Adapted with permission under the terms of the CC–BY–NC license from the Ref. [107]. Copyright 2023, the authors, published by Royal Society of Chemistry.

In 2023, a series of hemicyanine dyes (CyX–NEt_2_) containing different chalcogen atoms (X = O, S, and Se) were synthesized to achieve significant photophysical enhancements for efficient phototheranostics [107]. Figure 10 illustrates some of the relevant findings of that work. The Se atom substitution in the xanthene skeleton (CySe–NEt_2_) leads to red–shifted NIR absorption/emission (λ_abs_ = 776 nm, λ_em_ = 818 nm) spectra compared to those of O and S counterparts, as shown in Figure 10A. The heavy–atom effect leads to a decrease in the fluorescence quantum yield (Φ_f_ = 0.11) in CySe–NEt_2_, compared to those of CyS–NEt_2_ (Φ_f_ = 0.18) and CyO–NEt_2_ (Φ_f_ = 0.23). CySe–NEt_2_ exhibited a ^1^O_2_ generation yield of 14.8%, which is almost 15–fold more than its O analog. The PCE of CySe–NEt_2_ was determined to be 42.2%, which was notably higher than CyO–NEt_2_ (27.5%) and CyS–NEt_2_ (38.5%), displaying the role of Se atom substitution. Among the chalcogen–substituted hemicyanine dyes studied, CySe–NEt_2_ outperformed the other analogs in efficient PDT/PTT applications, despite its poor photostability, and enhanced dark cytotoxicity. To overcome these limitations, the hemicyanine backbone was also introduced with a clickable azide PEG chain group (CySe–mPEG_5K_). The amphiphilic CySe–mPEG_5K_ self-assembled into spherical NPs with a diameter of ∼100 nm in the aqueous medium, which is beneficial for tumor targeting via the EPR effect. Moreover, almost no change in the hydrodynamic diameter and TEM images in biological aqueous media confirmed the excellent chemical stability of NPs. Figure 10B highlights the self-assembly process of CyS–NEt_2_ and CySe–mPEG_5K_ and their implication in 4T1-tumor-bearing mice for multimodal phototheranostic application. The photophysical parameters remained unaltered for the fabricated CySe–mPEG_5K_ NPs and exhibited improved photothermal stability upon 750 nm laser irradiation. Additionally, this approach improves solubility and photostability, reduces dark cytotoxicity, and enhances targeting ability, making CySe–mPEG_5K_ NPs suitable for in vivo study. Figure 10C and 10D show the multimodal imaging of 4T1-tumorbearing mice treated with CySe–NEt_2_ and CySe–mPEG_5K_. However, CySe–mPEG_5K_ demonstrated superior NIR fluorescence/PAI signal in PBS, excellent photostability, fast/selective tumor accumulation, and low dark toxicity upon 750 nm laser irradiation. These findings accentuate the role of Se–decorated hemicyanine dyes in imaging-guided PDT/PTT combination therapy.

### 3.5. Selenophene–Fused Diketopyrrolopyrrole–Based TAs in Synergistic PDT/PTT

Diketopyrrolopyrrole derivatives have been widely used in paints, inks, organic solar cells, fluorescent sensors, and phototherapy applications due to their interesting photophysical properties [108]. On the other hand, the functionalization of selenophene groups in organic chromophores is one of the prominent strategies for designing efficient PSs and PTAs for synergistic phototherapy. In a similar fashion to thiophene, the presence of the selenophene group, further red shifts the absorption spectrum and augments the heavy-atom effect crucial for PTT and PDT effects. The rational design strategy, through selenophene functionalization in diketopyrrolopyrrole–based PSs, opens new directions for theranostic applications.

**Figure 11:**
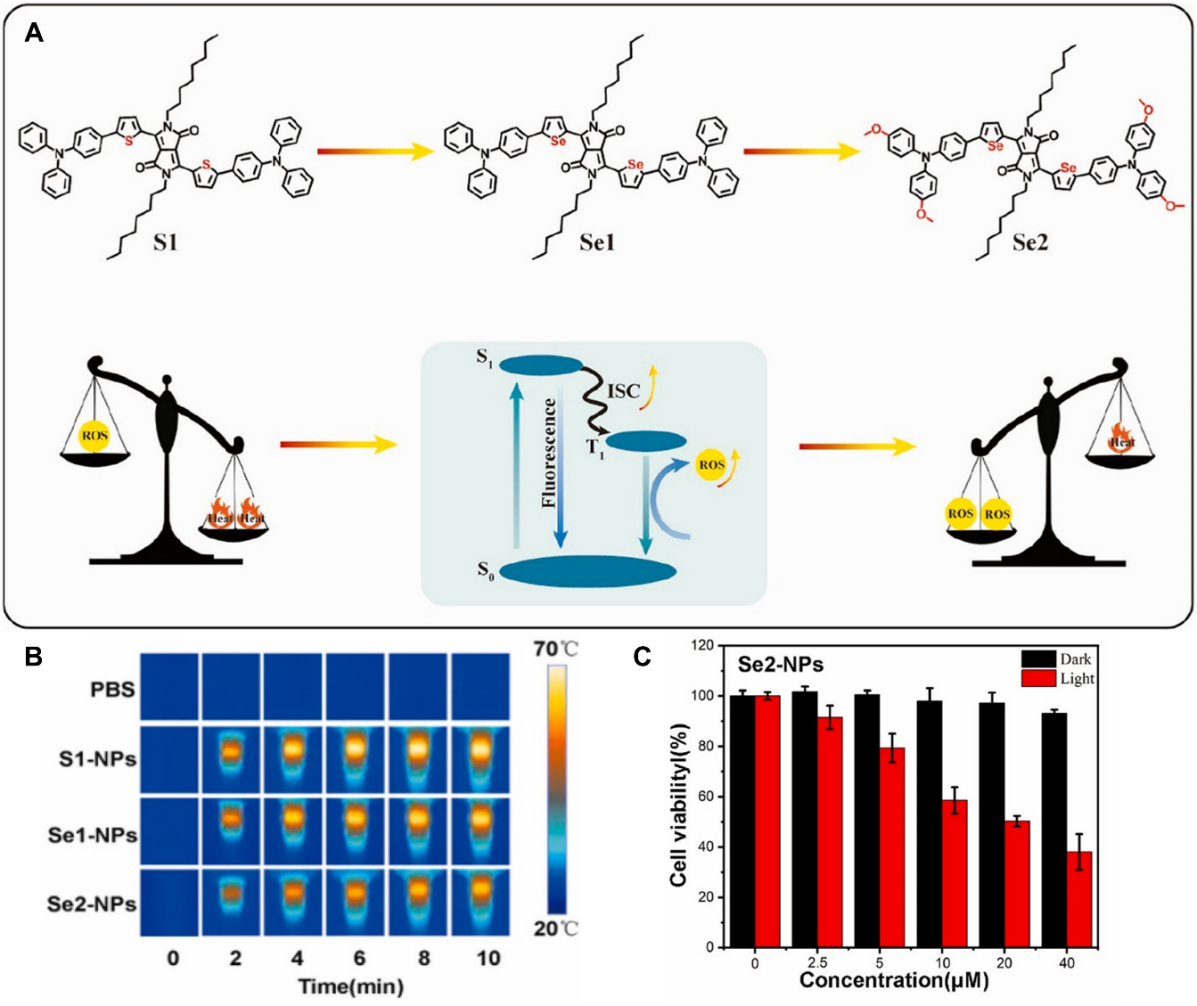
(**A**) Illustration of a rational design of diketopyrrolopyrrole–based compounds for efficient phototherapy. (**B**) IR thermal images of the 5 μM S1-NPs, Se1-NPs, and Se2-NPs under 635 nm laser irradiation at 0.3 W cm^−2^ for different times. (**C**) Relative viability of A549 cells treated with Se2-NPs at various doses under 635 nm laser irradiation or not. Adapted with permission from Ref. [109]. Copyright 2024, Elsevier.

Recently, in 2024, selective modification in the diketopyrrolopyrrole skeleton was performed with the thiophene group to construct a D–A structure (S1) [109]. In this work, the thiophene groups were replaced with selenophene groups (Se1) to enhance the heavy–atom effect, whereas a methoxy group was introduced to the triphenylamine moiety (Se2) to modulate the physicochemical and optical properties, as shown in Figure 11A. The UV–Vis absorption spectra of S1, Se1, and Se2 exhibit low-energy peaks at ~630, 650, and 660 nm, respectively, in THF solvent. The red shift in the absorption band is attributed to the looser delocalized electron cloud of the Se atom. The low fluorescence quantum yields of Se derivatives (Φ_f_ = 3.5% for Se1 and 2.9% for Se2) are lower than that of S1 (7.1%), suggesting an efficient ISC process in Se derivatives. This leads to ^1^O_2_ quantum yields of 0.28, 0.39, and 0.44 for S1, Se1, and Se2, respectively. To improve the water solubility and biocompatibility of the PSs, FDA–approved Pluronic F-127 was used to encapsulate S1, Se1, and Se2 to form S1-NPs, Se1-NPs, and Se2-NPs. DLS and TEM analyses revealed that the prepared NPs exhibit a spherical morphology with average hydrodynamic diameters of 200, 185, and 180 nm, respectively. The absorption spectra of all the NPs showed a red shift in the absorption compared to their molecular counterparts, indicating the aggregation behavior. The formed NPs resolved the solubility issue and displayed excellent photostability under 635 nm laser irradiation.

Figure 11B displays the IR thermal images for the studied NPs, in which the S1 NPs exhibit the highest PCE of 38.6%, which is opposite to the ROS yield order. Figure 11C shows the relative viability of A549 cells treated with Se2-NPs irradiated with a 635 nm laser, with an apoptosis rate of 52%. The Se2-NPs were chosen for in vivo experiments in the A549 tumor-bearing mice model. The in vivo studies in the mice model revealed that Se2-NPs derived from Se2 exhibited promising phototherapeutic performance via synergistic PDT/PTT against the A549 tumor. This demonstrates that the relative contributions of PDT and PTT can be fine–tuned by thiophene and selenophene functionalization in diketopyrrolopyrrole–based TAs to optimize their therapeutic efficiency.

In brief, Se is an extensively studied chalcogen element in PDT, PTT, and synergistic PDT/PTT applications. The literature survey reports show that Se atoms are integrated into organic molecules through exo/endocyclic ether O replacement and the functionalization of various functional groups, like S. The selenophene and selenodiazole groups are commonly derivatized with organic chromophores such as cyanine dyes, croconaine, BODIPY, diketopyrrolopyrrole, and other NIR–absorbing molecules. The Se substitution through these groups enhances the ICT effect, resulting in red–shifted NIR absorption and emission, which contributes to high PCE with PAI capability. Additionally, Se atoms induce a significant heavy–atom effect that enhances SOC and fosters near–unity triplet harvesting and ROS production. The combined ICT and SOC effects are stronger than those of their S analogs, making them promising candidates for PDT, PTT, and synergistic imaging-guided PDT/PTT applications. The nanoformulation of Se–containing TAs provides excellent photostability, effective prevention of unpleasant H–aggregation, biocompatibility, and selective tumor accumulation, resulting in better in vivo therapeutic performance. However, exocyclic seleno–carbonyl modification in organic molecules is still under investigation for phototherapy applications. Recent comparative studies have focused on the design and computational photophysical characterization of chalcogen–containing organic compounds, including psoralen, angelicin, naphthalimide, and Nile red dyes, which may lead to the development of high–performance Se–containing TA molecules [42,43,110,111,112].

## 4. Advances in Nano Delivery Strategies for Tellurium–Containing TAs in Phototherapy

Te is less explored than S and Se due to its rarity in living organisms and low abundance, rendering it a ‘forgotten’ chemical element [113]. However, Te derivatives are not toxic and exhibit exceptional biological activities, such as antioxidants and chemoprotective agents that are even more potent than the S and Se isosteres [18,23,114,115]. The incorporation of the Te atom into heterocycles and conventional chromophores leads to strong ICT and heavy–atom effects that are significantly more pronounced than those of the S and Se analogs. In the following, we discuss the advantages of the nanoformulation of Te–containing TAs, the key role of combining ICT and heavy–atom effects in optimizing PCE, and ROS production to enhance in vitro/in vivo PDT/PTT performances.

### 4.1. Chalcogenopyrylium–Fused Croconaine–Based PTAs in PTT

As discussed above, D–A–D–type croconaine (CR) dyes are excellent candidates for phototheranostic applications. On the other hand, chalcogenopyrilium dyes are also an important class of organic molecules possessing photochemotherapeutic applications [116,117]. The functionalization of the chalcogenopyrilium moiety in CR dye skeletons as the donor group bestows a new vision to modulate photophysical properties through selective chalcogen substitution.

**Figure 12:**
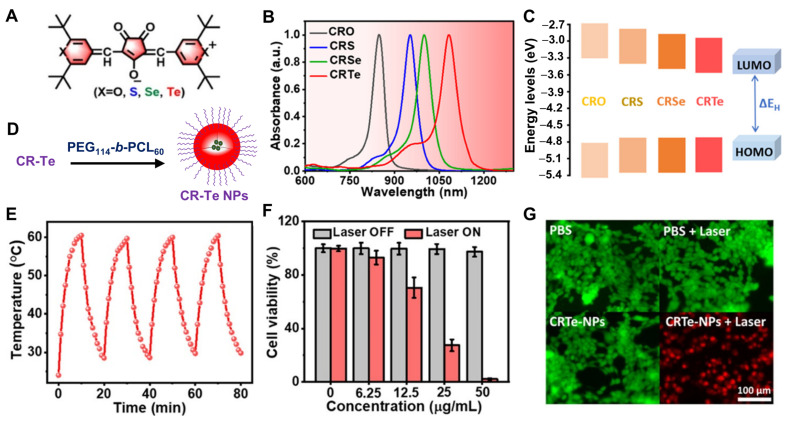
(**A**) Chemical structure of chalcogen atom–substituted CR dyes (CR-X, X = O, S, Se, and Te). (**B**) Absorption spectra of CRO, CRS, CRSe, and CRTe; (**C**) computed HOMO−LUMO energy gap of CRO, CRS, CRSe, and CRTe. (**D**) Nanoformulation strategy of CRTe. (**E**) Photothermal stability of CRTe NPs during four cycles of 1064 nm light irradiation. (**F**) Relative viability of 4T1 cells treated with various concentrations of CRTe NPs without or with 1064 nm laser irradiation (0.7 W cm^−2^). (**G**) Fluorescence microscope images of CRTe-NP-treated 4T1 cells co-stained with calcein–AM (green color, live cells) and propidium iodide (red color, dead cells). Adapted with permission from Ref. [27]. Copyright 2024, American Chemical Society.

In 2024, chalcogen–substituted CR dyes CR-X (X = O, S, Se, Te) were proposed for superior PTT application [27]. The important results of that work are highlighted below and illustrated in Figure 12.

The molecular structures of chalcogen–substituted CR dyes are shown in Figure 12A. Progressive heavier chalcogen incorporation into their structure red shifts the absorption intensity maximum to the NIR–II region, as shown in Figure 12B. Computational calculations revealed that the HOMO–LUMO energy gap decreases because of a change in the electronegativity of the heavy–atom–doped donor units promoting ICT, as depicted in Figure 12C. To improve the solubility and bioavailability, the CRTe molecule was encapsulated within amphiphilic PEG_114_–*b*–PCL_60_ to form polymeric NPs, as demonstrated in Figure 12D. The loading capacity and encapsulation efficiency were determined to be 19.7% and 98.5%, respectively. TEM and DLS studies confirmed the shape and size of the formed nanoaggregates, revealing an average diameter of 100 nm. The formed polymeric NPs showed excellent colloidal stability by maintaining a stable size distribution in water and 10% fetal bovine serum even after 7 days. The absorption spectrum of CRTe NPs exhibited a blue–shifted shoulder absorption band, indicating H–aggregation behavior. Additionally, the CRTe NPs demonstrated outstanding optoacoustic stability even for 30 min irradiation. The in vitro photothermal effect of CRTe NPs exposed to 1064 nm irradiation resulted in an almost 36.5 °C temperature increase with a PCE of 70.6%. Figure 12E displays the excellent photobleaching resistance of CRTe NPs upon four cycles of laser irradiation. Figure 12F shows the dose-dependent cell viabilities of the CRTe NPs in 4T1 breast cancer cells. The cell viability remains high across concentrations without laser irradiation, showing promising biocompatibility of CRTe NPs, whereas laser–induced heating leads to significant cell death. Moreover, Figure 12G visually demonstrates the photothermal damage of CRTe NPs–treated 4T1 cells with laser illumination, confirmed through a calcein–AM/propidium iodide (PI) assay. The in vivo study of 4T1 tumor-bearing mice upon 1064 nm laser irradiation showed efficient tumor eradication in mice. The overall finding of this study highlights the role of chalcogen atom substitution in designing PTAs for precise PTT–based cancer treatment.

### 4.2. Poly–Pyrrole Tellurium–Based Polymer NPs as TAs in Synergistic PDT/PTT

In past decades, organic semiconducting materials composed of π–conjugated building blocks have emerged as promising TAs for deep–tissue optical imaging, phototherapy, and photoactivation [118]. In the context of chalcogen compounds, thiophene–containing semiconducting polymeric TAs for PDT/PTT exhibit a superior tumor–suppressing effect [118,119,120,121]. However, the precise and systematic tuning of these nano–TAs in both hypoxic and normoxic environments had not been previously explored.

**Figure 13:**
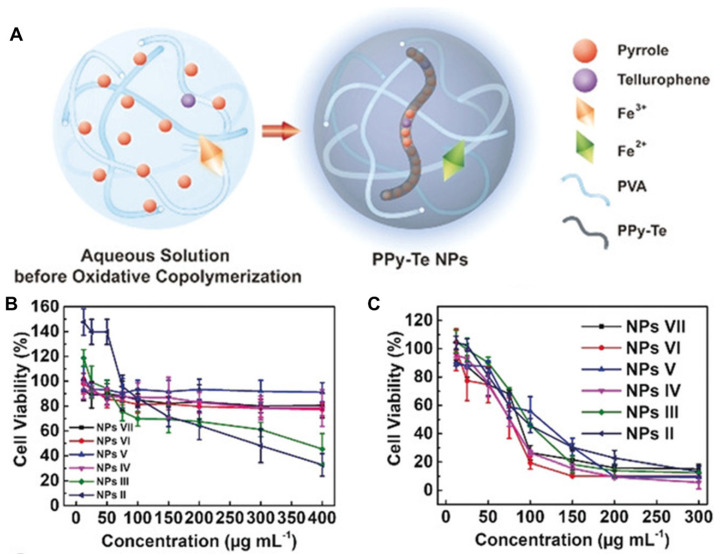
(**A**) Schematic illustration of the preparation of PPy–Te NPs; 4T1 cell viability when treated with NPs II–VII. (**B**) Cell viability in the dark and (**C**) upon 808-nm laser irradiation. Adapted with permission from Ref. [125]. Copyright 2020, John Wiley & Sons, Inc.

PPy NPs have been utilized previously in PTT due to their excellent PCE [122,123]. On the other hand, Te in tellurophene NPs can enhance SOC and promote ISC events, which is very important for efficient PDT [124]. In 2020, semiconducting PPy and tellurophene–based nano–TAs (PPy–Te NPs) were synthesized by controlling oxidative copolymerization with varied ratios of Py to tellurophene by FeCl_3_, as illustrated in Figure 13A [125]. Systematically, the NPs were prepared by adding Py and tellurophene to the molar ratio of 0/100 (NPs I), 85/15 (NPs II), 90/10 (NPs III), 92/8 (NPs IV), 95/5 (NPs V), 98/2 (NPs VI), and 100/0 (NPs VII), respectively. The TEM and DLS measurements confirmed the spherical shape of the fabricated NPs with sizes varying from 60 to 100 nm (NPs II–VII) with a small polydispersity index. This indicated the uniform size distribution of NPs suitable for enhanced permeability and the EPR effect. NPs IV demonstrated the highest stability, maintaining structural integrity even after one month of storage, as confirmed by zeta potential measurements.

The absorption intensity at 808 nm increases with the pyrrole content of NPs. The prepared NPs showed no fluorescence emission, suggesting dominant non–radiative relaxation processes. The presence of tellurophene in the NPs can enhance SOC and facilitate ISC for triplet–state generation, which is eventually responsible for ROS generation. The temperature for the series of PPy–Te NPs increased upon 808 nm laser irradiation for 10 min. This resulted in PCEs of 31.3%, 35.1%, 35.5%, 39.9%, 41.9%, and 43.6% for NPs II–VII, respectively. Furthermore, the degradation rate of DPBF upon increasing Te molar content was used to calculate ROS yields of 34.9%, 29.4%, 26.0%, 19.1%, 17.7%, and 15.9% for NPs II–VII, respectively. Figure 13B and 13C show the results of cell viability studies of NPs II–VII in the dark and upon 808 nm light irradiation, respectively. Under light, the cytotoxicity of NPs increased significantly, with the lowest EC_50_ value of 75.6 mg mL^−1^ for NPs IV. The precise regulation of photothermal and photodynamic effects in normoxic and hypoxic environments optimized the Py to tellurophene ratio 92/8 (NPs IV) with optimum PCE and ROS yield. NPs IV were chosen for in vivo experiments on 4T1 breast tumor-bearing female Balb/c mice model. The results displayed superior performance in inhibiting 4T1 tumor proliferation in the mice model, indicating the beneficial role of the Te-containing semiconducting NPs for synergistic phototherapy.

### 4.3. Chalcogen–Modified Iodinated BODIPY PSs/PTAs in NIR Phototherapy

**Figure 14:**
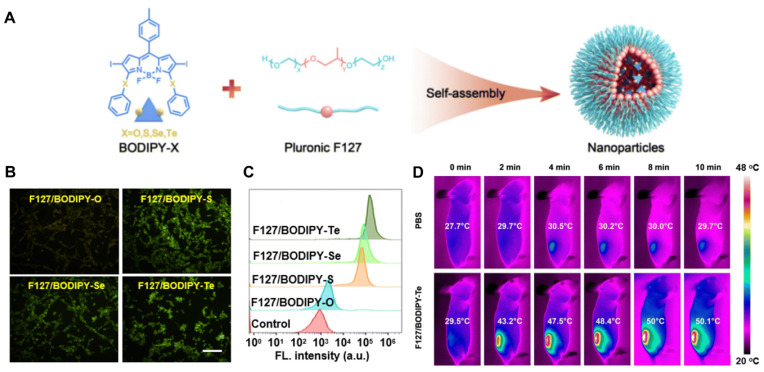
(**A**) Schematic illustration of the preparation process of F127/BODIPY-X (X = O, S, Se, and Te) NPs. In vitro study of U87 cells exposed to F127/BODIPY-X and 660 nm laser irradiation for 10 min via DCFH–DA. (**B**) Fluorescence microscopy images, scale bar: 100 μm, and (**C**) flow cytometry results of intracellular ROS generation. (**D**) IR thermal images of mice bearing U87 tumor cells treated with PBS or F127/BODIPY-Te NPs under irradiation of a 660 nm laser (1.0 W cm^−2^). Adapted from Ref. [26]. Copyright 2024, Royal Society of Chemistry.

Among the available NIR PSs, BODIPY derivatives have gained considerable attention due to their interesting photophysical properties [126]. Moreover, excellent photostability, low cytotoxicity, and good biocompatibility make BODIPY dyes attractive for NIR phototherapy [127,128]. In 2024, the NIR phototherapeutic effect of chalcogen–substituted iodinated BODIPY-X (X = O, S, Se, and Te) derivatives was investigated [26]. Figure 14 illustrates the main findings of that study. The electronic donating ability of heavier chalcogens enhanced the ICT event and lowered the HOMO–LUMO energy gap, leading to a red–shifted absorption from 558 nm, 610 nm, and 618 nm to 660 nm by incorporating O, S, Se, and Te, respectively. The fluorescence quantum yields of BODIPY-X were determined to be 8.9%, 2.1%, 1.2%, and 0.01% for O, S, Se, and Te derivatives, respectively. The decrease reflects the heavy–atom effect from iodine and heavier chalcogens facilitating efficient ISC. BODIPY-X was encapsulated within Pluronic–F127, an amphiphilic block copolymer, through a nanoprecipitation method, as demonstrated in Figure 14A. The drug loading efficiencies of F127/BODIPY-O, F127/BODIPY-S, F127/BODIPY-Se, and F127/BODIPY-Te NPs were 3.31%, 1.70%, 7.40%, and 1.24%, respectively. DLS analysis revealed that the average hydrodynamic diameters of F127/BODIPY-X NPs were about 238.2, 196.3, 160.4, and 100.8 nm, respectively, which correlated well with the sizes observed in the TEM images. The produced F127/BODIPY-X composite NPs showed enhanced hydrophilicity and bioavailability suitable for therapeutic application. In vitro cytotoxicity studies on human glioblastoma (U87) cells were performed via 2′,7′–dichlorodihydrofluorescein diacetate as a probe for ^1^O_2_ generation. The bright fluorescent images upon a 660 nm laser irradiation for 10 min confirmed the ROS generation for F127/BODIPY-X NPs with excellent cellular uptake (Figure 14B). Figure 14C displays the flow cytometry results, which showed the most intense fluorescence signal for cells treated with F127/BODIPY-Te NPs. The results show a stronger ROS generation capability than other composites. The significant photothermal effect of these NPs under NIR irradiation raises the temperature of tumor sites to above 50 °C.

Figure 14D displays IR thermal images showing a pronounced tumor ablation effect on U87-bearing mice, validating the PDT/PTT and imaging–guided superior therapeutic efficacy of BODIPY-Te in vivo. In summary, Te derivatives are the least explored TAs in PDT, PTT, and synergistic PDT/PTT applications. The reported studies demonstrate exo/endocyclic O replacement by Te in various organic chromophores such as BODIPY, croconaine, and polymeric derivatives. Overall research on Te atom incorporation into organic molecules shows pronounced ICT, resulting in redshifted NIR absorption and emission bands that significantly exceed those of S and Se analogs. Compounds having a Te atom, being heavier than S and Se ones, exhibit enhanced heavy–atom effects, leading to efficient ISC processes and triplet–state generation, and subsequently, larger ROS yields. The nanoformulation of Te–containing TAs further improves photostability, solubility, and bioavailability without compromising their promising ICT and SOC effects. Therefore, Te–containing TAs demonstrate significant in vitro and in vivo therapeutic efficacy against various tumors compared to their S and Se analogs.

In biomedical applications, the above studies signify that the redox–modulating properties of organoselenium and organotellurium compounds are particularly scrutinized for designing the necessary PSs and PTAs for cancer phototherapy. However, the collateral impact of Se, Te, and Se/Te–containing TAs on physiological functions must be considered, particularly their tendency to interfere with redox-cellular balance, induce oxidative stress by disrupting antioxidant defense, and cause off–target toxicity [19,21,129,130,131]. Se is heavier and more polarizable than S, and Se derivatives possess lower oxidation potentials, enabling them to participate more readily in redox reactions [17,94]. Thus, it is important to maintain the level of Se–containing TAs, since elevated doses could lead to pro–oxidant activity, resulting in increased toxicity and several side effects to human health [131,132,133,134]. On the other hand, the use of Te and Te–containing TAs is gaining gradual attention among the scientific community and is less explored [23,114,115]. Though Te is the heaviest non radioactive chalcogen atom and chemically similar to the others, it is not essential to the human body. Its toxicity depends upon the chemical form, the dose, and the physiological functions [135,136]. Thus, the careful optimization of dosage, in vitro and in vivo biocompatibility, and target–specific delivery are crucial to overcome such toxicological effects while preserving therapeutic efficacy.

## 5. Conclusions and Future Directions

In summary, this feature review highlights the advancements made over the last five years in nanoformulation strategies for heavier chalcogen (S, Se, and Te)–substituted PSs and PTAs used in anticancer PDT, PTT, and synergistic PDT/PTT applications. This review outlines and discusses the fundamental principles of PDT/PTT, the design principles of chalcogen–based PSs and PTAs, their key spectroscopic features, and the pivotal role of the nanoformulation strategy in efficient delivery, ROS production, and heat generation, enhancing therapeutic capabilities. The heavier chalcogen-containing TAs (PSs and PTAs) meet all necessary criteria for PDT and PTT and offer an excellent opportunity for multimodal imaging–guided combinatorial therapy. During the last five years, chalcogen atoms like S, Se, and Te were incorporated into well–known organic chromophores such as BODIPY, PDI, croconaine, porphyrin, cyanine dyes, diketopyrrolopyrrole, polymeric derivatives, metal complexes, and other NIR–II–absorbing molecules through exo/endocyclic functionalization. Chalcogen incorporation red–shifted the absorption and emission into the VIS–NIR region due to enhanced ICT. Heavier chalcogen substitution induces a strong SOC–mediated heavy–atom effect, promoting efficient ISC and near-unity triplet–state quantum yields for improved ROS generation. S is the most widely incorporated element and explored chalcogen for phototherapeutic applications. Conversely, Te–containing compounds possess the most suitable photophysical properties for synergistic PDT/PTT. The precise regulation of the chalcogen substitution approach and tailored nanoformulation strategies to control in vitro and in vivo ROS production and heat generation for PDT/PTT were discussed, along with selected examples. The nanoprecipitation and nanoencapsulation of chalcogen–containing TAs using polymer, silica, and micellar carriers promisingly improve their delivery and biological performances. The most significant outcomes from the reported studies demonstrate that the nanoformulation techniques significantly mitigate the existing issues associated with chalcogen–containing TAs by preventing H–aggregation and improving bioavailability, biocompatibility, photostability, and selective tumor accumulation ability. This improves the in vitro and in vivo efficacy of chalcogen–containing TAs.

Last but not least, preliminary nanotechnological approaches, such as employing polymeric micelles and polymeric NPs, lipid NPs, and mesoporous silica NPs to encapsulate chalcogen–containing TAs, lay the groundwork for designing and making effective nano-drug delivery systems. This can only be achieved by engineering nanocarriers of appropriate size, biocompatibility, and specificity, and improved targeting ability. The use of nanocarriers such as carbon dots, nanotubes, dendrimers, nanocomposite hydrogels, metal–organic frameworks, covalent–organic frameworks, and hydrogen–bonded organic frameworks for target–specific in vitro/in vivo studies of chalcogen compounds has not been explored sufficiently for cancer phototherapy. As light is the first key to trigger and control the related processes, further detailed photophysical characterization of nanoformulated PSs and PTAs is necessary to modulate ROS generation and PCE. The efficient design, photophysical characterization, and nano delivery of seleno– and telluric–carbonyl modified TAs for in vitro/in vivo studies have yet to be explored. Moreover, the parallel development of new chalcogen–containing PSs and PTAs is essential to enhance the nanoencapsulation process and their phototherapeutic efficiency. We anticipate that this review will open new avenues in the design, synthesis, spectroscopic insights, and cellular study applications of nanoformulated chalcogen–containing TAs for cancer phototherapy.

## Abbreviations

The following abbreviations are used in this manuscript:

AIEgens	Aggregation–induced emission luminogens
D-A	Donor–acceptor
DPBF	1,3-diphenylisobenzofuran
DLS	Dynamic light scattering
HAFPSs	Heavy–atom free photosensitizers
HOMO	Highest occupied molecular orbitals
ICT	Intramolecular charge transfer
ISC	Inter–system crossing
LUMO	Lowest unoccupied molecular orbitals
NIR	Near infrared
NPs	Nanoparticles
PAI	Photoacoustic imaging
PBS	Phosphate buffer solution
PCE	Photothermal conversion efficiency
PDT	Photodynamic therapy
PEG	Polyethylene glycol
PSs	Photosensitizers
PTAs	Photothermal agents
PTT	Photothermal therapy
ROS	Reactive oxygen species
^1^O_2_	Singlet oxygen
SEM	Scanning electron microscopy
SOC	Spin–orbit coupling
ΔE_ST_	Singlet–triplet energy gap
TD-DFT	Time–dependent density functional theory
TEM	Transmission electron microscopy
VIS	Visible
VIS-NIR	Visible to NIR
